# Habitat segregation and migration in tropical anguillid eels, *Anguilla bengalensis bengalensis* and *A. bicolor bicolor*

**DOI:** 10.1038/s41598-020-72788-9

**Published:** 2020-10-09

**Authors:** Takaomi Arai, Inn-Ju Chai, Yoshiyuki Iizuka, Chih-Wei Chang

**Affiliations:** 1 Environmental and Life Sciences Programme, Faculty of Science, Universiti Brunei Darussalam, Jalan Tungku Link, Godoing, 1410 Brunei Darussalam; 2 Institute of Tropical Aquaculture, Universiti Malaysia Terengganu, 21030 Terengganu, Malaysia; 3 Institute of Earth Sciences, Academia Sinica, Taipei, 11529 Taiwan; 4 National Academy of Marine Research, Kaohsiung, 80661 Taiwan; 5 Department of Oceanography, National Sun Yat-Sen University, Kaohsiung, 80424 Taiwan; 6 National Museum of Marine Biology and Aquarium, Pintung, 94450 Taiwan

**Keywords:** Ecology, Zoology

## Abstract

Anguillid eels of the genus *Anguilla*, which have a unique catadromous life history, are widely distributed across many parts of the world. However, little research has been conducted on the behavioural mechanisms of habitat segregation between sympatric species in tropical anguillid eels. To understand the ecological and behavioural mechanisms involved in the life history and migration of tropical anguillid eels, strontium (Sr):calcium (Ca) ratios were examined in otoliths of *A. bengalensis bengalensis* (41 specimens) and *A. bicolor bicolor* (130 specimens) collected from ten rivers in northwestern Peninsular Malaysia. The otolith Sr:Ca ratios revealed different habitat use between the two species. The broad range of otolith Sr:Ca ratios and habitat shift found in *A. bicolor bicolor* suggested that its habitat utilization was opportunistic in environments of varying salinity. *A. bicolor bicolor* prefers to live in the midstream to downstream areas with tidal influences. *A*. *bengalensis bengalensis*, however, was found to only reside in freshwater environments throughout their continental growth. *A*. *bengalensis bengalensis* tends to live in upstream area with no tidal influence. Their habitat use, migratory history, and habitat distribution indicate that habitat segregation occurs between the two species, leading to the different habitat preferences in tropical river systems.

## Introduction

The anguillid eels, genus *Anguilla* Schrank, are widely distributed across the globe. There are nineteen species and subspecies of the freshwater eels, thirteen of which live in tropical areas^1,2^. Of these thirteen species/subspecies, seven species/subspecies are found in Indonesia and Malaysia in the western Pacific^1,2^. As catadromous fishes, anguillid eels spend most of their lives in freshwater until they return to their spawning grounds in the tropics, although part of the population never enters freshwater and instead resides in brackish and marine areas close to coastlines^3^. There are five developmental stages in freshwater eels: leptocephalus (larval), glass eel and elver (juveniles), yellow eel (immature adult) and silver eel (mature adult). Leptocephali are passively transported through oceanic currents, metamorphose into glass eels and consequently locate within continental habitats for 4–8 months after hatching^4^. After the continental habitats, glass eels and elvers become yellow eels and settle in rivers and lakes. Yellow eels undergo a metamorphosis into silver eels with gonadal maturation during autumn and winter, after which the temperate eels consequently start a spawning migration to the ocean. In tropical eels, however, the period of downstream migrations extends throughout the year^4–7^. The year-round spawning and stable larval development stimulate year-round recruitment to continental habitats in tropical eels^8–12^, leading to significantly different life history characteristics between temperate and tropical anguillid species.

West Malaysia (Peninsular Malaysia) is located in the southern half of the Malay Peninsular and is divided from East Malaysia (Borneo Island) by the South China Sea between the Pacific and Indian Oceans; it is believed to be one of the most important geographical niches for anguillid eels. Recently, the tropical eels, *Anguilla bengalensis bengalensis*, *A. bicolor bicolor* and *A. marmorata* have been reported in northwest Peninsular Malaysia^7,13–18^. The reproductive characteristics of *A. bicolor bicolor* and *A. bengalensis bengalensis* were examined, leading to findings of the unique year-round downstream migration in the tropical waters. There has been little research, however, on their life history, migratory patterns, habitat usage and spatial distribution in tropical ecosystems. Unlike temperate habitats, multiple species live sympatrically in tropical ecosystems, potentially complicating the life history, migratory patterns as a result of inter- and intraspecific competition. The tropical species are believed to be closer to the basal form than the temperate species. Research on the migration and life history of tropical anguillid eels has revealed the origin of catadromy in anguillid eels and how the thousands of kilometres of large-scale migration in temperate eels have been established in contrast to the few hundred kilometres of local migration in tropical eels^19^.

Analysis of the otolith microchemistry is broadly used to trace of the migratory history of the temperate eels *A. anguilla*^20–23^, *A. rostrata*^24,25^, *A. japonica*^26–29^ and *A. australis* and *A. dieffenbachaii*^30^ and the tropical eels *A. marmorata*^28,31–35^, *A. bicolor pacifica*^31,36^, *A. bicolor bicolor*^36–38^ and *A. mossambica*^33^. The concentration of Sr in seawater is approximately 100 times higher than freshwater. A positive correlation was found between Sr concentrations in the otolith and Sr:Ca ratios and salinity in freshwater eels^39–41^. Sr concentrations and Sr:Ca ratios in otoliths fluctuate depending on the durations the eels lived in freshwater or seawater. The otolith Sr:Ca ratios were strongly influenced by the salinity of the ambient water, while other factors, such as water temperature, food and physiological factors were found to minimally affect the ratios in the Japanese eels^39,40^. Therefore, Sr:Ca ratios from core to edge reflect the salinity of the eel’s habitat throughout its lifetime^3^. Although these studies found that some anguillid eels migrated to a freshwater environment in the growth phase while others did not, the reasons for the discrepancy why some eels at growth phase did not migrate to freshwater still remain unclear.

In the present study, we examined the migratory history and habitat use of the tropical freshwater eels, *Anguilla bengalensis bengalensis* and *A. bicolor bicolor* in West Malaysia. The objectives of this study were to (1) reconstruct the migratory history of tropical anguillid eels by examining otolith Sr:Ca ratios along the life history transect, (2) estimate the Sr:Ca ratios in otolith edge parts to determine recent habitat use and (3) examine the habitat preference, species-specific migratory behaviours and habitat segregation between these two species.

## Results

### Species composition and distribution

**Figure 1:**
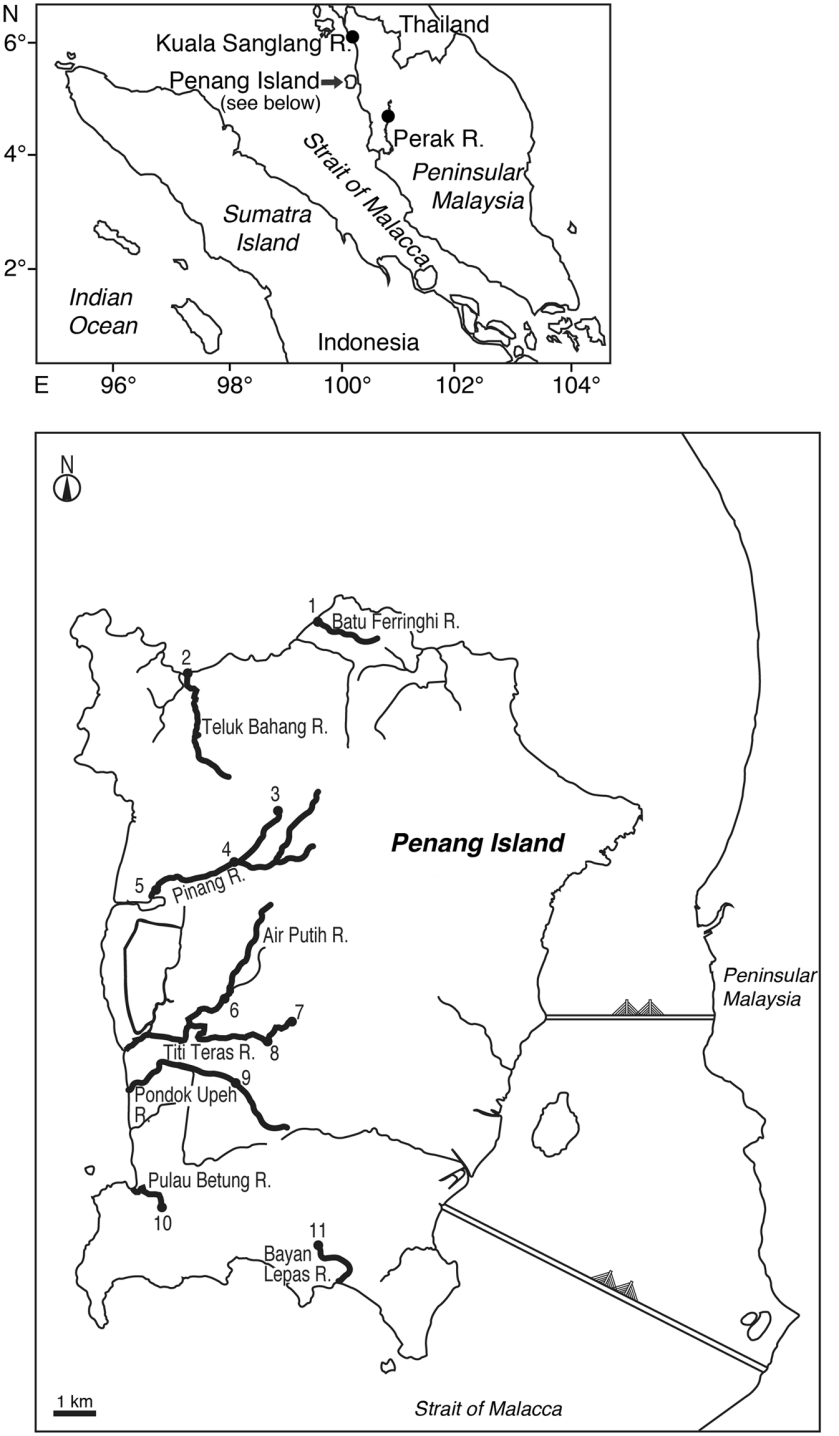
Sampling sites for tropical anguillid eels in northwest Peninsular Malaysia. Specimens were caught in one site in the Kedah State (downstream area of the Kuala Sanglang River), one site in the Perak State (upstream are of the Perak River) and 11 sites in eight rivers on Penang Island (Penang State). In Penang Island, 1, Batu Ferringhi in the downstream area of the Batu Ferringhi River; 2, Teluk Bahang in the downstream area of the Teluk Bahang River; 3, Titi Kerawang Waterfall in the upstream area of the Pinang River; 4, Kampung Sungai Pinang in the midstream area of the Pinang River; 5, Kuala Sungai Pinang in the downstream area of the Pinang River; 6, Air Putih in the midstream area of the Air Putih River; 7, Titi Serong in the upstream area of the Titi Teras River; 8, Titi Teras in the midstream area of the Titi Teras River; 9, Pondok Upeh in the midstream area of the Pondok Upeh River; 10, Pulau Betung in the midstream area of the Pulau Betung River; 11, Bayan Lepas in the midstream area of the Bayan Lepas River. The map was traced by the author using Adobe Illustrator CS6 and referring to Google Maps 2016 (https://maps.google.com/).

A total of 171 specimens of the two tropical anguillid eels, *Anguilla bengalensis bengalensis* (41 specimens) and *A. bicolor bicolor* (130 specimens) were collected from ten sites in the northwestern part of Peninsular Malaysia (Fig. 1). Numbers of *A. bengalensis bengalensis* were highest in the upstream (94.9%, n = 37) while the numbers of *A. bicolor bicolor* were highest in the midstream (95.8%, n = 92) and downstream (100.0%, n = 36) of the rivers (Table 1). *A. bicolor bicolor* was the only species found in downstream regions. Both eel species were found to coexist in the upstream and midstream regions of the Titi Teras River, and in the midstream of the Pinang and Bayan Lepas Rivers (Table 1).

**Table 1:** Number of anguillid eels collected in each site of the rivers in northwest Peninsular Malaysia.

River	Upstream		Midstream		Downstream	
	*A. bengalensis*	*A. bicolor*	*A. bengalensis*	*A. bicolor*	*A. bengalensis*	*A. bicolor*
	*bengalensis*	*bicolor*	*bengalensis*	*bicolor*	*bengalensis*	*bicolor*
Kuala Sanglang River	–	–	–	–	–	8
Batu Feringhi River	–	–	–	–	–	1
Teluk Bahang River	–	–	–	–	–	3
Pinang River	22	–	1	2	–	24
Air Putih River	–	–	–	4	–	–
Titi Teras River	6	2	2	72	–	–
Pondok Upeh River	–	–	–	1	–	–
Pulau Betung River	–	–	–	4	–	–
Bayan Lepas River	–	–	1	9	–	–
Perak River	9	–	–	–	–	–

no eel.

All *A. bengalensis bengalensis* specimens were found mainly in upstream locations with no tidal effect, where salinity ranged from 0.01 to 0.03 psu, water temperatures ranged from 23.6 to 28.1 °C and elevation in each river was higher (Table 1, 2). *A. bicolor bicolor* were found from downstream to midstream with tidal effects ranging from 0.01 to 27.8 psu, temperatures were warmer (even up to or greater than 30 °C), and a lower elevation existed in each river (Table 1, 2).

**Table 2:** Environmental parameters and topography of the rivers in northwest Peninsular Malaysia where anguillid eels were collected.

River	Location	Distance from estuary (km)	Elevation (m)	Depth (m)	Water temperature (°C)	Salinity (psu)	Tidalinfluence
**Kedah State**							
Kuala Sanglang River	D	3.4	6	0.5–1.5	35.7	0.21	No
**Penang State**							
Batu Feringhi River	D	0.3	2–8	0.1–0.5	26.1–26.7	0.06–0.09 ( low tide)	Yes
Teluk Bahang River	D	0.6	2–10	0.1–0.8	27.5–28.5	0.04–1.93 ( low tide)	Yes
						27.8 ( high tide)	
Pinang River	U	5.2	174–217	0.2–2.5	23.6–24.2	0.01	No
Pinang River	M	4.7	36–38	0.2–2.0	27	0.01	No
Pinang River	D	1.6	4–11	0.5–1.5	26.7–29.9	0.25–0.31	Yes
Air Putih River	M	5.1	14–15	0.5–1.5	26.3	0.02	No
Titi Teras River	U	7.9	50–75	0.2–0.5	25.8	0.02	No
Titi Teras River	M	4.9	19–23	0.5–2.0	26.5	0.02	No
Pondok Upeh River	M	5.8	13–15	0.1–0.5	28	0.03	No
Pulau Betung River	M	0.9	5–10	0.5–1.0	30.2	0.15 ( low tide)	Yes
Bayan Lepas River	M	2.3	12–14	0.5–1.0	26.9	0.03	No
**Perak State**							
Perak River	U	197	41	7.0–13.0	28.1	0.01	No

*U* upstream, *M* midstream, *D* downstream.

### Size, age and relative growth

The TLs of 92.0% of *A. bengalensis bengalensis* (n = 37) found in upstream regions ranged from 375.0 to 1162.0 mm with mean ± standard deviation (SD) of 695.7 ± 216.5 mm; and the BWs ranged from 65.8 to 5100.0 g (954.6 ± 1040.5 g). The ages of *A. bengalensis bengalensis* specimens ranged from 3 to 9 years (6.1 ± 1.7 years) (Table 3).

**Table 3:** Biological characteristics of the anguillid eels used in the present study.

Species, rivers	Sex	Number	Total length (mm)		Body weight (g)		Age (years)	
			Mean ± SD	Range	Mean ± SD	Range	Mean ± SD	Range
***A. bengalensis bengalensis***								
Pinang River	F	11	681.2±135.8	375–920	701.5 ± 465.1	65.8–1700	5.9 ± 1.1	4–8
	M	1	504	–	56.7	–	5	–
	I	1	565	–	316.3	–	3	–
	U	8	478.6±91.3	375–610	211.2 ± 134.9	71.9–445	4.9 ± 1.1	4–6
Titi Teras River	F	5	576.6±112.7	431–768	383.4 ± 218.5	100.0–762.7	5.3 ± 1.3	4–7
	M	3	427.7±50.0	357–464	145.0 ± 67.0	51.8–205.9	5.0 ± 1.4	4–6
Bayan Lepas River	F	1	726	–	772.4	–	6	–
Perak River	F	11	985.3±84.1	885–1162	2305.3 ± 1086.2	1376.6–5100.0	8 ± 1.1	6–9
***A. bicolor bicolor***								
Kuala Sanglang	F	8	636.4±52.8	568–700	559.5 ± 177.3	362–824	6.3 ± 0.7	5–7
Batu Feringhi River	M	1	392	–	118.8	–	3	–
Teluk Bahang River	F	2	529.0±50.9	493–565	249.7 ± 77.6	194.8–304.5	5.5 ± 0.7	5–6
	U	1	383	–	65.7	–	4	–
Pinang River	F	24	597.5±73.6	455–755	407.1 ± 202.7	174.8–1124.7	5.9 ± 0.7	5–7
	M	2	388.0±6.0	382–394	122.3 ± 1.5	120.8–123.8	2.5 ± 0.5	2–3
Air Putih River	F	4	567.8±52.6	506–613	385.0 ± 197.8	256.9–677.2	6.0 ± 0.8	5–7
Titi Teras River	F	42	482.9±131.8	219–695	247.7 ± 178.7	17.3–607.8	4.5 ± 1.0	2–7
	M	28	342.4±65.4	234–558	67.6 ± 29.1	18.8–127.8	3.1 ± 0.7	2–5
	I	4	253.2±27.8	211–287	25.3 ± 8.2	14.1–37.1	3 ± 0.7	2–4
Pondok Upeh River	M	1	380	–	106.9	–	3	–
Pulau Betung River	F	4	565.8±17.4	540–577	342.1 ± 50.1	28.3–495.6	5.3 ± 1.0	4–6
Bayan Lepas River	F	9	584.6±67.4	444–679	419.2 ± 119.5	183.3–609.7	5.6 ± 1.2	3–7

*SD* standard deviation, *F* female, *M* male, *I* intersex, *U* unknown.

The TLs of 95.8% of *A. bicolor bicolor* (n = 92) found in midstream regions ranged from 211.0 to 695.0 mm (442.4 ± 137.4 mm), the BWs ranged from 14.1 to 677.2 g (204.3 ± 180.7 g) and the ages ranged from 2 to 7 years (4.3 ± 1.3 years old). One hundred percent of the *A. bicolor bicolor* specimens were found in downstream regions (n = 36); TLs ranged from 382.0 to 755.0 mm (581.5 ± 96.2 mm), BWs ranged from 65.7 to 112.5 g (404.8 ± 222.3 g), and the age ranged from 2 to 7 years (5.7 ± 1.2 years) (Table 3).

The length–weight relationships were BW = 4.1TL–1923.6 in *A. bengalensis bengalensis* and BW = 1.4TL–396.5 in *A. bicolor bicolor*. *A. bengalensis bengalensis* was found to be slightly heavier than *A. bicolor bicolor* at the same length.

### Life history transects

The otolith Sr:Ca ratios along the life history transect showed the same patterns around the central region in all specimens. An abrupt increase in Sr:Ca ratios (11.7 × 10^**−**3^**–**15.7 × 10^**−**3^) was found approximately 150 μm from the otolith core, which corresponds to the elver mark.

**Figure 2:**
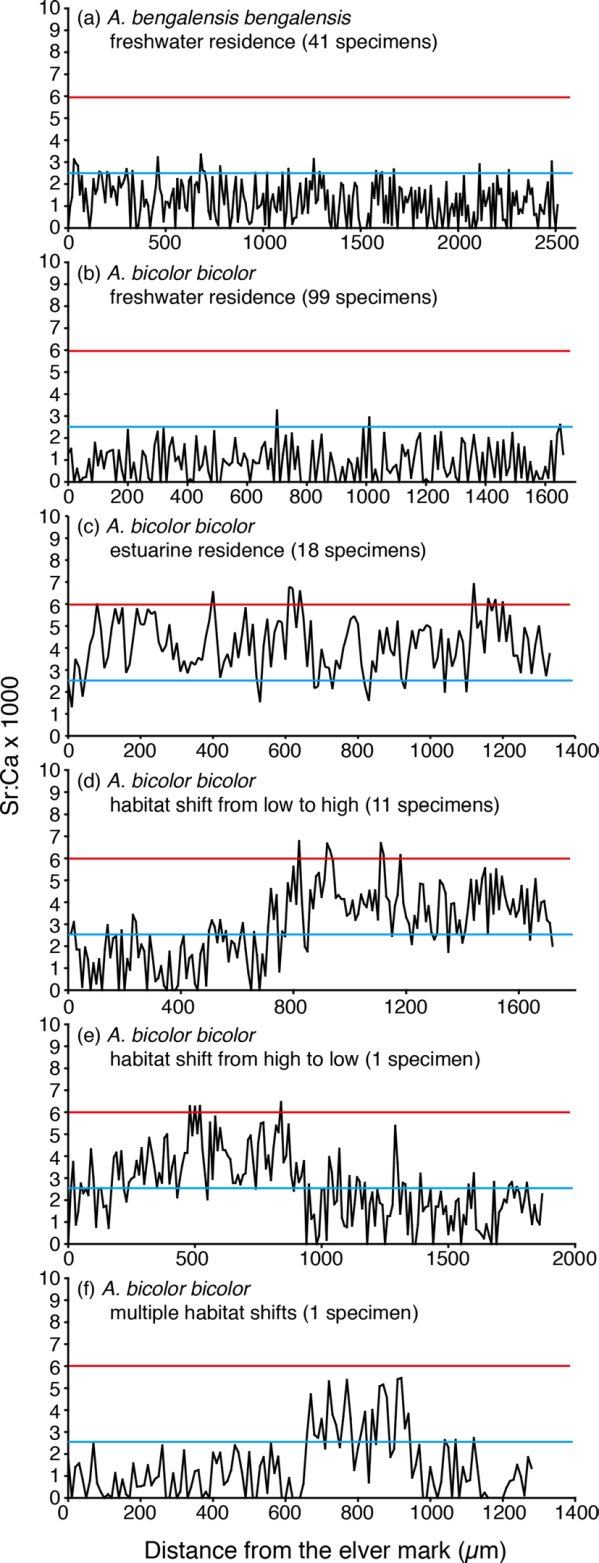
Migratory history of tropical anguillid eels, *Anguilla bengalensis bengalensis* and *A. bicolor bicolor*. Representative plots of otolith Sr:Ca ratios along a transect line from the elver mark to the edge of the otolith for *A. bengalensis bengalensis* (**a**) and *A. bicolor bicolor* (**b–f**). The red and blue lines in each panel indicate the marine water life period (≥ 6.0 × 10^−3^ in Sr:Ca ratios) and the freshwater life period (≤ 2.5 × 10^−3^ in Sr:Ca ratios), respectively.

After the elver mark, otolith Sr:Ca ratios along the life history transect differed between *A. bengalensis bengalensis* and *A. bicolor bicolor* (Fig. 2). Otolith Sr:Ca ratios in *A. bicolor bicolor* were highly varied, while little variation was found in the ratios of *A. bengalensis bengalensis* (Table 4).

**Table 4:** Mean and edge otolith Sr:Ca ratios and migratory patterns and recent habitat as determined from the otolith Sr:Ca ratios in anguillid eels in northwest Peninsular Malaysia.

Species	Location	Sex	Maturation	Number of specimens	Age (years)		Mean Sr:Ca ratios		Migratory	Edge Sr:Ca ratios		Recent	Number
			Stage		Mean ± SD	Range	Mean ± SD	Range	Patterns	Mean ± SD	Range	Habitat	
*A. bengalensis* *bengalensis*	Perak River (upstream)	All	All	11	8.0 ± 1.1	6–9	1.40 ± 0.50	0.69–2.48	Freshwater	1.08 ± 0.65	0.30–2.21	Freshwater	11
		F	II	3	7.5 ± 0.7	7–8	0.98 ± 0.25	0.69–1.15	Freshwater	0.53 ± 0.51	0.30–1.12	Freshwater	3
		F	III	2	–	8–9	–	1.22	Freshwater	–	0.51–0.97	Freshwater	2
		F	IV	4	7.3 ± 1.5	6–9	1.39 ± 0.34	1.18–1.89	Freshwater	1.30 ± 0.53	0.77–2.01	Freshwater	4
		F	V	2	–	9	–	1.94–2.48	Freshwater	–	1.41–2.21	Freshwater	2
	Pinang River (upstream)	All	All	19	5.4 ± 1.3	3–8	1.44 ± 0.41	0.75–2.17	Freshwater	1.20 ± 0.94	0.00–2.48	Freshwater	19
		F	I	6	5.7 ± 1.0	4–7	1.61±0.31	1.14–2.01	Freshwater	1.14 ± 1.08	0.00–2.24	Freshwater	6
		F	III	4	6.3 ± 1.5	5–8	1.17 ± 0.11	1.09–1.33	Freshwater	0.90 ± 0.85	0.00–1.88	Freshwater	4
		F	IV	1	–	6	–	0.95	Freshwater	–	2.31	Freshwater	1
		U	N/A	8	4.6 ± 1.2	3–6	1.52 ± 0.50	0.75–2.17	Freshwater	1.26 ± 0.94	0.00–2.48	Freshwater	8
	Pinang River (midstream)	M	II	1	–	5	–	1.99	Freshwater	–	1.51	Freshwater	1
	Titi Teras River (upstream)	All	All	7	5.2 ± 1.2	4–7	2.21 ± 0.25	1.83–2.52	Freshwater	1.54 ± 0.94	0.00–2.46	Freshwater	7
		F	I	3	4.7 ± 0.6	4–5	2.21 ± 0.33	1.83–2.40	Freshwater	1.57 ± 1.36	0.00–2.46	Freshwater	3
		F	II	2	–	7	–	2.00–2.08	Freshwater	–	0.88–0.94	Freshwater	2
		M	I	1	–	4	–	2.52	Freshwater	–	1.96	Freshwater	1
		M	II	1	–	6	–	2.24	Freshwater	–	2.33	Freshwater	1
	Titi Teras River (midstream)	All	All	2	–	4–6	–	0.76–1.50	Freshwater	–	0.00	Freshwater	2
		F	I	1	–	6	–	1.50	Freshwater	–	0.00	Freshwater	1
	Bayan Lepas River (midstream)	M	I	1	–	4	–	0.76	Freshwater	–	0.00	Freshwater	1
		F	III	1	–	6	–	0.76	Freshwater	–	0.00	Freshwater	1
*A. bicolor bicolor*	Batu Feringhi (downstream)	M	III	1	–	3	–	1.81	Freshwater	–	6.21	Marine	1
	Teluk Bahang River (downstream)	All	All	3	5.0 ± 1.0	4–6	1.32 ± 0.55	0.87–1.60	Freshwater	–	0.00–0.74	Freshwater	2
											6.64	Marine	1
		F	II	2	–	5–6	–	0.88–1.94	Freshwater	–	0.74	Freshwater	1
											6.64	Marine	1
		U	N/A	1	–	4	–	1.14	Freshwater	–	0.00	Freshwater	1
	Pinang River (midstream)	F	III	2	–	5–6	–	1.40–1.44	Freshwater	–	1.47	Freshwater	1
											2.57	Estuarine	1
	Pinang River (downstream)	All	All	21	5.7 ± 1.3	2–7	1.52 ± 0.45	0.96–2.09	Freshwater	0.84 ± 0.91	0.00–2.17	Freshwater	18
		All	All	3	5.7 ± 0.6	5–6	2.63 ± 0.06	2.56–2.67	Estuarine	3.92 ± 0.85	3.07–5.29	Estuarine	5
											8.39	Marine	1
		F	I	1	–	5	–	1.67	Freshwater	–	3.76	Estuarine	1
		F	III	8	5.8 ± 0.7	5–7	1.69 ± 0.51	0.96–2.47	Freshwater	0.95 ± 0.98	0.00–2.17	Freshwater	7
			III	1	–	6	–	2.67	Estuarine	–	3.42–4.07	Estuarine	2
		F	IV	4	6.8 ± 0.5	6–7	1.52 ± 0.67	1.04–2.48	Freshwater	1.08 ± 1.25	0.00–2.27	Freshwater	4
			IV	2	–	5–6		2.56–2.66	Estuarine	–	5.29	Estuarine	1
										–	8.39	Marine	1
		F	V	6	6.0 ± 0.7	5–7	1.41 ± 0.25	1.12–1.74	Freshwater	0.43 ± 0.60	0.00–1.44	Freshwater	5
											3.07	Estuarine	1
		M	III	2	–	2–3	–	1.12–1.17	Freshwater	–	1.29–2.20	Freshwater	2
	Air Putih River (midstream)	F	III	2	–	6–7	–	1.18–1.43	Freshwater	–	0.00	Freshwater	2
		F	IV	1	–	5	–	1.75	Freshwater	–	2.63	Estuarine	1
		F	IV	1	–	6	–	3.36	Estuarine	–	3.28	Estuarine	1
	Titi Teras River (upstream)	F	III	2	–	4	–	1.49–2.50	Freshwater	–	0.00–1.31	Freshwater	2
	Pulau Betung River (midstream)	F	III	2	–	4–6	–	1.55–1.67	Freshwater	–	2.20	Freshwater	1
											4.95	Estuarine	1
		F	IV	1	–	6	–	1.23	Freshwater	–	0.00	Freshwater	1
		F	V	1	–	6	–	1.92	Freshwater	–	2.67	Estuarine	1
	Bayan Lepas River (midstream)	F	All	9	5.6 ±1.3	3–7	1.11 ± 0.18	0.84–1.12	Freshwater	1.08 ± 0.81	0.00–2.23	Freshwater	7
										–	2.71–4.08	Estuarine	2
		F	II	2	–	3–6	–	1.14–1.18	Freshwater	–	1.39–2.23	Freshwater	2
		F	III	3	6.5 ± 0.7	6–7	1.26 ± 0.09	1.17–1.35	Freshwater	–	0.00–1.25	Freshwater	2
											2.71	Estuarine	1
		F	IV	3	6.0 ± 1.0	5–7	1.00 ± 0.19	0.89–1.22	Freshwater	–	0.00–1.43	Freshwater	2
											4.08	Estuarine	1
		F	V	1	–	5	–	0.88	Freshwater	–	1.27	Freshwater	1
	Titi Teras River (midstream)	All	All	67	3.8 ± 1.1	2–6	1.22 ± 0.43	0.63–2.34	Freshwater	1.13 ± 0.78	0.00–2.46	Freshwater	54
										3.45 ± 0.62	2.69–4.64	Estuarine	13
		All	II, III, IV	5	5.6 ± 1.1	4–7	3.17 ± 0.62	2.63–4.17	Estuarine	–	2.02	Freshwater	1
										3.22 ± 0.42	2.77–3.77	Estuarine	4
		F	I	5	3.6 ± 1.1	2–5	1.49 ± 0.53	1.01–2.11	Freshwater	1.55 ± 0.53	0.68–2.11	Freshwater	5
		F	II	4	4.0 ± 0.8	3–5	0.95 ± 0.21	0.77–1.25	Freshwater	1.00 ± 0.70	0.00–1.57	Freshwater	4
		F	III	15	4.6 ± 0.9	3–6	1.31 ± 0.47	0.71–2.27	Freshwater	1.24 ± 0.95	0.00–2.77	Freshwater	11
										4.07 ± 0.56	3.55–4.64	Estuarine	4
		F	III	3	6.0 ± 1.0	5–7	2.85 ± 0.33	2.63–3.23	Estuarine	3.03 ± 0.23	2.77–3.22	Estuarine	3
		F	IV	10	4.6 ± 0.7	4–6	1.51 ± 0.59	0.76–2.34	Freshwater	1.48 ± 0.66	0.70–2.03	Freshwater	4
										3.29 ± 0.45	2.80–3.93	Estuarine	6
		F	IV	1	–	6	–	3.15	Estuarine	–	2.02	Freshwater	1
		F	V	2	–	4	–	0.69–1.51	Freshwater	–	0.00–1.63	Freshwater	2
		M	I	11	2.9 ± 0.5	2–4	1.18 ± 0.32	0.85–1.94	Freshwater	1.22 ± 0.79	0.00–2.07	Freshwater	10
										–	3.35	Estuarine	1
		M	II	11	3.5 ± 0.7	3–5	1.07 ± 0.21	0.84–1.59	Freshwater	1.10 ± 0.89	0.00–2.46	Freshwater	10
										–	2.69	Estuarine	1
		M	II	1	–	4	–	4.17	Estuarine	–	3.77	Estuarine	1
		M	III	5	2.8 ± 0.8	2–4	0.95 ± 0.10	0.87–1.10	Freshwater	1.10 ± 0.84	0.00–2.36	Freshwater	5
		U	N/A	4	2.8 ± 0.5	2–3	1.10–0.34	0.76–1.48	Freshwater	0.47 ± 0.64	0.00–1.36	Freshwater	4
	Pondok Upeh River (midstream)	M	III	1	–	3	–	1.38	Freshwater	–	0.50	Freshwater	1
	Kuala Sanglang River (downstream)	All	II, III, IV	8	6.3 ± 0.8	5–7	1.28 ± 0.34	0.73–1.83	Freshwater	1.73 ± 0.62	1.00–2.25	Freshwater	4
										3.74 ± 1.23	2.95–5.16	Estuarine	4
		F	II	2	–	6	–	1.33–1.83	Freshwater	–	1.43–2.25	Freshwater	2
		F	III	5	6.4 ± 0.9	5–7	1.16 ± 0.28	0.73–1.40	Freshwater	–	1.00–2.25	Freshwater	2
										3.74 ± 1.23	2.95–5.16	Estuarine	3
		F	IV	1	–	6	–	2.66	Estuarine	–	3.40	Estuarine	1

*F* female, *M* male, *U* unknown.

In *A. bengalensis bengalensis*, the life history migratory patterns of all specimens from the six sites in 5 rivers resulted in consistently low Sr:Ca ratios (range, 0.7**–**2.5 × 10^**−**3^) along the life history transect (Fig. 2a). The lowest otolith Sr:Ca ratios were found in the silver eels of IV and V with ages of 6**–**9 years (7 specimens), suggesting that these eels lived their continental lives in a freshwater environment just before their downstream migration to open ocean (Table 4).

The variations in the otolith Sr:Ca ratios of *A. bicolor bicolor* were also divided into three types. The first pattern showed consistently low Sr:Ca ratios (0.6**–**2.5 × 10^**−**3^) along the life history transect (n = 99) (Fig. 2b). The second pattern showed the estuarine type (n = 18), with consistently higher Sr:Ca ratios (2.6–4.2 × 10^−3^) throughout the life history transect (Fig. 2c). The third pattern showed a switch type (n = 13) that was further classified into three categories (Fig. 2d-f). The first switch type showed a change in Sr:Ca ratios from a low phase averaging 0.8**–**2.0 × 10^−3^ approximately 290–1330 µm from the otolith core to a high phase, averaging 2.6**–**4.3 × 10^−3^ the otolith edge (11 of 13 specimens) (Fig. 2d). Significant differences were found between the low Sr:Ca ratio phase and the high Sr:Ca ratio phase (t-test, t = 2.6050**–**13.4417, df = 139**–**219, *p* < 0.01**–**0.0001). The second switch type showed Sr:Ca ratios changing from a high phase averaging 3.6 × 10^−3^ approximately 930 µm from the otolith core to a low phase averaging 1.7 × 10^−3^ at the otolith edge (1 of 13 specimens) (Fig. 2e). A significant difference was found between the high Sr:Ca ratio phase and the low Sr:Ca ratio phases (t-test, t = 9.3207**–**12.1663, df = 61**–**93, *p* < 0.0001). The third switch type showed multiple shifts in habitats (Fig. 2f). One specimen showed a low Sr:Ca ratios phase averaging 0.9 × 10^−3^ approximately 650 µm from the otolith core, then the ratios increased to an average of 3.5 × 10^−3^ from 650 to 940 µm from the otolith core, and finally the ratio decreased to an average of 0.8 × 10^−3^ at the otolith edge (Fig. 2f). Among the 14 matured eels aged 5–7 years in stages IV and V, four eels were found to have had an estuarine residence and ten eels were found to have had a freshwater residence throughout their lives (Table 4).

### Habitat use

The average Sr:Ca ratios (± SD) outside of the elver mark in *A. bengalensis bengalensis* collected from six sites were 1.4 ± 0.5 × 10^−3^ (11 specimens in the upstream of the Perak River), 1.4 ± 0.4 × 10^−3^ (19 specimens in the upstream of the Pinang River), 2.0 × 10^−3^ (1 specimen in the midstream of the Pinang River), 2.2 ± 0.3 × 10^−3^ (7 specimens in the upstream of the Titi Teras River), 0.8**–**1.5 × 10^−3^ (2 specimens in the midstream of the Titi Teras River) and 0.8 × 10^−3^ (1 specimen in the midstream of the Bayan Lepas River) (Table 4). Although all *A. bengalensis bengalensis* specimens from all sites were categorized as having a freshwater residence, significant differences were found in the average Sr:Ca ratios between the upstream of the Titi Teras River and the upstream of the Perak and Pinang rivers (ANOVA, N = 7**–**19, Q = 5.7062**–**5.8966, *p* < 0.01). No significant differences were found between the upstream of the Perak River and the upstream of the Pinang River (ANOVA, N = 11**–**19, Q = 0.4007, *p* > 0.05).

Sr:Ca ratios in the otolith edges of *A. bengalensis bengalensis* from six sites were ranged from 0.0 to 2.5 × 10^−3^ (Table 4). The Sr:Ca ratios in the otolith edge indicated that the recent habitat before capture in all *A. bengalensis bengalensis* specimens was in freshwater environments. There were no significant differences in Sr:Ca ratios in the otolith edges for all combinations of the upstream of the Perak and Pinang rivers and upstream of Titi Teras River (ANOVA, N = 7**–**19, Q = 0.5070**–**1.5636, *p* > 0.05).

Based on the average Sr:Ca ratios outside of the elver mark in *A. bicolor bicolor*, revealed that there were specimens of both freshwater and estuarine residences (Table 4). The freshwater residence was found in the downstream of the Batu Feringi River (1.8 × 10^−3^ in 1 specimen), in the downstream of the Teluk Bahang River (1.3 ± 0.6 × 10^−3^ in 3 specimens), in the midstream of the Pinang River (1.4 × 10^−3^ in 2 specimens), in the upstream of the Titi Teras River (1.5**–**2.5 × 10^−3^ in 2 specimens), in the midstream of the Pulau Betung River (1.6**–**1.7 × 10^−3^ in 2 specimens), in the midstream of the Bayan Lepas River (1.1 ± 0.2 × 10^−3^ in 9 specimens) and in the midstream of the Pondok Upeh River (1.4 × 10^−3^ in 1 specimen). There were sympatric freshwater and estuarine residences in the downstream of the Pinang River, in the midstream of the Air Putih River, in the midstream of the Titi Teras River and in the downstream of the Kuala Sanglang River (Table 4). The average Sr:Ca ratios of freshwater residences in the downstream of the Pinang River, the midstream of the Air Putih River, the midstream of the Titi Teras River and the downstream of the Kuala Sanglang River were 1.5 ± 0.5 × 10^−3^ (n = 21), 1.8 × 10^−3^ (n = 1), 1.2 ± 0.4 × 10^−3^ (n = 67) and 1.3 ± 0.3 × 10^−3^ (n = 7), respectively. The average Sr:Ca ratios of the estuarine residences in the downstream of the Pinang River, the midstream of Air Putih River, the midstream of the Titi Teras and the downstream of the Kuala Sanglang River were 2.6 ± 0.1 × 10^−3^ (n = 3), 3.4 × 10^−3^ (n = 1), 3.2 ± 0.6 × 10^−3^ (n = 5) and 2.7 × 10^−3^ (n = 1), respectively. Significant differences in the average Sr:Ca ratios were found between the freshwater and estuarine residences in the downstream of the Pinang River and the midstream of the Titi Teras River (t-test, t = 4.1497**–**9.5361, df = 22**–**70, *p* < 0.0005**–**0.0001).

Sr:Ca ratios in the otolith edges of *A. bicolor bicolor* from eleven sites ranged from 0.0 to 8.4 × 10^−3^ (Table 4). Interestingly, the Sr:Ca ratios in the otolith edge indicated that the recent habitat was either freshwater, brackish water or marine water environments in *A. bicolor bicolor*. In downstream sites of the Batu Feringhi, Teluk Bahang, and Pinag rivers, the recent marine environment habitats led to Sr:Ca ratios more than 6.0 × 10^−3^, although their average Sr:Ca ratios outside of the elver mark indicated either freshwater or estuarine residences (Table 4). Similar discrepancies between the Sr:Ca ratios in the otolith edge and the average Sr:Ca ratios were found in the midstream of the Pinang River (1 of 2 specimens), the Air Putih River (1 of 2 specimens), the Pulau Betung River (1 of 2 specimens), the Bayan Lepas River (2 of 9 specimens), the Titi Teras River (13 of 67 specimens) and in the downstream of the Kuala Sanglang River (4 of 8 specimens) (Table 4).

## Discussion

A systematic study of the diversity and distribution of tropical freshwater eels in West Malaysia was performed in a previous study^18^. Approximately 500 eels were found in the northwestern peninsular area; *A. bicolor bicolor* was the dominant species (88.1%), *A. bengalensis bengalensis* was the second most common (11.7%), and *A. marmorata* was the least common species (0.2%). *A. bicolor bicolor* was distributed widely from the upstream to the downstream areas in each river, although it was more abundant in the downstream and midstream regions, and it was rarely found in upstream areas. *A. bengalensis bengalensis*, however, preferred to live mainly in the upstream and rarely occurred in midstream areas^18^. That study did not examine the eels’ migratory history or habitat use throughout their lives. Anguillid eels have diverse migration patterns between freshwater and marine habitats^3^, and therefore, sampling sites during field observation might not necessarily reveal the exact habitats and movements of the fish.

Reconstruction of migratory history by means of otolith Sr:Ca ratios has revealed switching between environments with different levels of salinity in a number of anguillid eels^3^. This study is the first report on the migratory history and habitat use of *A. bengalensis bengalensis*. *A. bengalensis bengalensis* in northwest Peninsular Malaysia was only found in freshwater environments in the migration patterns, corresponded to the filed observations of the upstream to the midstream areas with no tidal influences^18^. Although the otolith Sr:Ca ratios in *A. bengalensis bengalensis* differed between sites, the values still correlated with the freshwater value of less than 2.5 × 10^−3^. The difference in otolith Sr:Ca ratios even within freshwater environments would differ slightly due to regional (river) water chemistry. The life history transect of the otolith Sr:Ca ratios showed no habitat shifts during their lives remaining freshwater environments in *A. bengalensis bengalensis*. *A. bengalensis bengalensis* would move to upstream or midstream immediately without tidal influence after recruitment into the estuary and settle in the freshwater environment for 6–9 years until starting their downstream migration to the open ocean. Unlike *A. bengalensis bengalensis*, *A. bicolor bicolor* was found to have diverse migration, i.e., both freshwater and estuarine residences. *A. bicolor bicolor* was also found to shift habitat to either freshwater, brackish water or marine water during their lives. The diverse migratory history and habitat use corresponded to the distribution range from downstream to upstream as found in the previous study^18^. Such diverse migratory behaviours and habitat use were also found in *A. bicolor bicolor* in Indonesia^36–38^ and in *A. bicolor pacifica* in the Philippines^31^ and Vietnam^34^.

Wide varieties of migratory history and habitat use were found in *A. bicolor bicolor*, although the preferred degree of salinity and migratory patterns differed from those found in other countries. Although 76% of *A. bicolor bicolor* had freshwater residence and 24% had estuarine residence in this study, 100% of the species had either estuarine or marine residences with no freshwater residence in a coastal lagoon of Indonesia^36,37^ and estuarine or marine residences were also used in 100% of *A. bicolor pacifica* with no freshwater residence in a river in Vietnam^31^. There was 23% in freshwater residence and 77% was in estuarine or marine residences in *A. bicolor pacifica* in the Philippines^31^. *A. bicolor bicolor* and *A. bicolor pacifica* prefer to live in higher salinity environments except for northwest Peninsular Malaysia, where there are multiple eel species living sympatrically in tropical river systems.

Intraspecific environmental habitat preference or interspecific competition may lead differences in migratory pattern and habitat use for each species. In Vietnam and the Bonin and Amami islands of Japan, 93%, 52% and 100% of *A. marmorata* were estuarine residents^32,34,35^, although more than 70% of *A. marmorata* had freshwater residence in the Philippines and Taiwan^31^. There were *A. bicolor pacifica* and *A. marmorata* in the Philippines, *A. marmorata* and *A. japonica* in Taiwan and *A. bicolor pacifica* and *A. marmorata* in Vietnam, while only *A. marmorata* occurred in a river system in the Bonin and Amami islands of Japan. *A. marmorata* in the Bonin and Amami islands can migrate from marine to freshwater environment with no interspecific competition, although the habitat preference differed between these two sites in *A. marmorata*.

In a coastal lagoon area of Indonesia, the migratory patterns of *A. bicolor bicolor* were mainly of the switch type shifting from freshwater to seawater environments (75%) with the other eels lived uniformly in brackish water (5%) or seawater (20%)^37^. *A. bicolor bicolor* was only found in the lagoon of Indonesia^37^, although other anguillid eels, *A. bengalensis bengalensis* and *A. marmorata* were also in the water system. Habitat segregation due to interspecific competition might occur in the Indonesian lagoon area. After recruitment, *A. bicolor bicolor* would migrate upstream of the rivers and settle for certain periods. Thereafter, most of the *A. bicolor bicolor* population would move back downstream into the lagoon due to the interspecific competition among the three species.

In northwest Peninsular Malaysia, *A. bengalensis bengalensis*, *A. bicolor bicolor* and *A. marmorata* were found. While the dominant species was *A. bicolor bicolor* (88%), *A. bengalensis bengalensis* was found mainly in upstream of rivers and mostly segregated from the distribution of *A. bicolor bicolor*. Thus, *A. bicolor bicolor* might live and migrate from downstream to upstream in various levels of salinity in northwest Malaysia. Indeed, 10% of *A. bicolor bicolor* underwent habitat shifts during their lives and 17% of the specimens changed their habitats within a short period as found in the Sr:Ca ratios of the otolith edge. Ratios of sympatric species, environmental habitat preference including salinity in each species and interspecies competition might determine the habitat use and migration patterns of tropical anguillid eels. Habitat use might be opportunistic and is also susceptible to inter- and intraspecific variations in each distribution range.

Mean and edge otolith Sr:Ca ratios suggested that *A. bengalensis bengalensis* perpetually lived in freshwater environments in all rivers, although a discrepancy was found between the mean and edge Sr:Ca ratios in the otolith in *A. bicolor bicolor* from six rivers. Although mean Sr:Ca ratios in otolith showed both freshwater and estuarine residences, the Sr:Ca ratios in the otolith edge showed further marine residence values more than 6.0 × 10^−3^ in *A. bicolor bicolor*. These results suggest that *A. bicolor bicolor* might frequently change habitat, or move to habitats with different salinity for some periods, and could therefore live in freshwater, brackish water or marine water environments during their lives.

In northwest Peninsular Malaysia, *A. bengalensis bengalensis* preferred to live mainly in the upstream areas with freshwater environments, lower water temperatures and higher elevation (Table 1, 2). In contrast, *A. bicolor bicolor* preferred to live more downstream to midstream with tidal influences, warmer temperatures and lower elevation (Table 1, 2). Nevertheless further field observation measuring various environmental factors are needed, as these results demonstrate some habitat segregation between *A. bengalensis bengalensis* and *A. bicolor bicolor* within river systems in northwest Peninsular Malaysia. Two New Zealand eels, *A. australis* and *A. dieffenbachii*, lived in high salinity, while *A. australis* showed higher salinity preference in a lake^30^. These results suggested differences in habitat use between these two species. *A. australis* was caught more often by the eel fishery in the lake, while fewer *A. dieffenbachii* were caught^42^. *A. dieffenbachii* was found more often in the upper part of the estuary than *A.australis* which uniformly was more abundant in the estuary^43^. It was suggested that *A. dieffenbachii* prefers to live in faster flowing water with, larger sized substrates and in upstream areas, while *A. australis* dominates in lowland lakes, marshes and slower flowing stretches of streams and rivers^44–46^. Therefore, habitat segregation can occur through water systems in anguillid eels in sympatric habitats, although each species could have ecological plasticity, and their distributions appear to often overlap.

In the matured *A. bicolor bicolor* eels in stages IV and V, 62.5% eels lived in the same habitats continuously either freshwater (54.2%) or estuary (8.3%), while 37.5% of the eels changed their habitats from either freshwater to brackish water (33.3%) or from brackish water to freshwater (4.2%) before starting their downstream migration to the open ocean (Table 4). All matured *A. bengalensis bengalensis* eels, however, were found to have a freshwater residence throughout their lives. Most of the eels did not change their salinity habitats after settlement in each habitat.

Migratory history and habitat use in *A. bicolor bicolor* in nothwest Peninsular Malaysia differed from those of *A. bicolor bicolor* in a lagoon in the central Java of Indonesia, where 75% of eels showed a habitat shift from freshwater to marine water as mentioned above^37^. Temperate eels of the American and New Zealand eels have restricted home ranges and territories^47–51^. The current results suggest that tropical anguillid eels generally have home ranges and territories and move short distances in each river. However, intraspecific difference in migratory behaviours might occur depending on biotic and abiotic factors such as habitat environments and interspecific competition at each site.

Age at maturation of *A. bengalensis bengalensis* and *A. bicolor bicolor* ranges from 6 to 9 years and from 4 to 7 years, respectively (Table 3, 4). Timing of maturation overlapped but occurs later in *A. bengalensis bengalensis* than in *A. bicolor bicolor. A. bengalensis bengalensis* lives in the upstream of rivers with lower temperature than *A. bicolor bicolor*, which lives in downstream areas with higher temperature. Furthermore, growth patterns and maturation size also differ between the two species. Differences in habitat environment and growth may lead to a difference in the timing of maturation. Ages at maturation of the temperate anguillid eels, *A. anguilla*, *A. rostrata*, *A. japonica*, *A. australis* and *A. diefenbachii* ranged from 6–50 years^52^, 5–22 years^53^, 5–17 years^54^, 15–40 years^55^ and 10–64 years^56^, respectively. These results suggest that age at maturation of tropical eels is earlier than in temperate eels. Compared with temperate region, water temperatures in Malaysia (28 °C) are higher and more stable throughout the year^6^. The consistent and warm tropical climate might induce the different timing of maturation between the tropical and temperate eels.

## Methods

### Fish

A total of 171 anguillid eels were collected in different riverine regions of ten sites from the states of Kedah (Kuala Sanglang River), Penang (Batu Feringhi River, Teluk Bahang River, Pinang River, Air Putih River, Titi Teras River, Pulau Betung River, Bayan Lepas River and Pondok Upeh River in Penang Island) and Perak (Perak River) in the northwestern part of Peninsular Malaysia (Fig. 1) from May 2014 to July 2016. Downstream of the Kuala Sanglang River in Kedah State was approximately 3.4 km from an estuary. Rising tide could be influenced and controlled by irrigation gates with a depth range of 0.5–1.5 m and an elevation of 6 m. Penang Island is composed of an area of approximately 300 km^2^, and all rivers are shorter and narrower compared to those in the mainland. Elevation is highest at upstream in the Pinang River (174–217 m), followed by upstream in the Titi Teras River (50- 75 m) and midstream in the Pinang River (36–38 m). The Perak River is the largest river in the mainland of Peninsular Malaysia at approximately 400 km in length.

Sampling location details such as distance from an estuary, elevation and depth and water parameters such as salinity and water temperature were measured (Table 2). Salinity ranged from 0.01–0.21 psu for all sampling sites without tidal effect but it was slightly higher at tidal areas which were downstream in the Batu Feringhi River (0.06–0.09 psu at low tide), midstream in the Pulau Betung River (0.15 psu at low tide), downstream in the Pinang River (0.25–0.31 psu at low tide) and downstream in the Teluk Bahang River (0.04–1.93 psu at low tide and 27.8 psu at high tide). Water temperature ranged from 23.6 to 35.7 °C for all sampling sites (Table 2).

All specimens were collected at night using hooks and lines or eel traps. All specimens were frozen immediately after collection and purchased from local fishermen. Our protocols followed the ethical guidelines for the use of animals of Universiti Malaysia Terengganu (UMT) and were approved by the animal ethics committee at UMT. Total length (TL) and body weight (BW) were measured, and sex was determined by visual and gonadal histology^7^. The maturation stage of all eels were referred from Arai and Abdul Kadir^7^. Maturation stages correspond to a growth phase (stages I and II), a pre-migrant phase (III) and two migrating phases (IV and V) in female. In male, maturation stages correspond to immature (stage I), early maturation (stage II) and mid-maturation (stage III) stages. Species identification was performed by both methods of morphological analysis^14–16^ and molecular genetic analysis using mitochondrial cytochrome oxidase subunit I (COI) and 16S ribosomal RNA (16S rRNA) sequence^16^.

### Otolith preparation and microchemistry analysis

Both sagittal otoliths were extracted and cleaned in an ultrasonic bath and rinsed with distilled water. Otoliths were placed in sealed Eppendorf tubes and left to dry at room temperature. Next, the otoliths were embedded in silicon rubber moulds, filled with epofix resin (with the distal side facing upwards) and left to dry in an oven at 53 ºC for 8 h. Excessive epofix resin was removed using a low-speed circular saw (Beuhler Metaserv, Buehler, UK Ltd.). Samples were first ground using coarser wet grinding paper (P2400), followed by finer wet grinding paper (P1200) with the use of a Buehler Metaserv grinder-polisher (Buehler, UK Ltd.). Otoliths were further polished with aluminium oxide 0.05 μm on polishing paper. The process of grinding and polishing continued until the core of the otoliths was revealed. Thereafter, the otoliths were periodically rinsed in running water and checked under a compound light microscope to ensure the samples were not over ground. Next, the samples were arranged on round glass plates, and the otoliths were coated with carbon to obtain the electrical conductivity. Line-transect analyses of the otoliths were conducted using an electron probe micro analyser (EPMA, JEOL JXA-8900R) with beam conditions of 15 kV for the acceleration voltage, 3nA for the beam current, 5 × 4 µm for the rectangular scanning beam size and a spot interval of 10 µm. Calcite (CaCO_3_) and strontianite (SrCO_3_) were used as standards to calibrate the concentration of calcium and strontium, respectively, in the otoliths. Wavelength dispersive spectrum at the strontium Lα peak position was measured for 80 s at the peak and for 20 s for each of the upper and lower baselines. Ca was measured for 20 s at the Kα peak and for 10 s at both sides of the baseline^57^. The elver mark forming the core of the otolith corresponds to the leptocephalus and early glass eel stages during oceanic life^58^. Therefore, the otolith Sr:Ca ratios during oceanic life were omitted to analysis migratory pattern and habitat use.

### Age determination

After EPMA analyses, all otoliths were etched with 5% EDTA for approximately 1 min to reveal the annual rings for age determination^28,31^. The otoliths were observed under a compound light microscope and the ages of the eels were determined by the number of annual rings evident on the otoliths. The criteria which were determined the otolith annuli in temperate eels were adapted in this study and were also used in tropical eels such as *A. marmorata*^28,31^ and *A. bicolor pacifica*^31^. The distinct transition check in the otoliths, which is thought to be associated with the entrance into freshwater habitats, corresponded to an age of 0 years^28,31^. It was considered that one pair of translucent and opaque rings corresponds to 1 year and that the ages of the tropical eels was estimated by counting the translucent rings outside the elver mark^28,31^. False annuli were distinguished from supernumerary check based on their width, optical density, relative positions and degree of continuity around the otolith circumference^31,59,60^. However, difficulties in counting the number of rings did arise due to the presence of false annuli; therefore, only countable samples were used in this study to avoid otolith annuli interpretation error.

### Classification of migratory pattern and habitat use

Several specific otolith Sr:Ca ratios are used as the benchmark for a freshwater life history, although, these values vary between regions and authors^3,22,25,26,35–37^. Different researchers have used different Sr:Ca ratios to differentiate a freshwater environment, such as < 2.0 × 10^−3^^32^, < 2.5 × 10^−3^^34^ and < 4.0 × 10^−3^^28,31,33^. In this study, specimens of *A. bengalensis bengalensis* from upstream of the Pinang River, which is free from tidal influences, were selected to represent the range of Sr:Ca ratios in the otoliths of freshwater eels. No specimens of *A. bicolor bicolor* were found in the upstream of this river. Since otolith Sr:Ca ratios of *A. bengalensis bengalensis* were all ≤ 2.5 × 10^−3^ (freshwater residence), this value was used to differentiate a freshwater environment from brackish or marine environment in the present study. The value used to determine a seawater environment was ≥ 6.0 × 10^−3^ (marine residence), as documented in previous studies in anguillid eels^3,26^. The brackish water environment was then calculated as (2.5 × 10^−3^ < Sr:Ca < 6.0 × 10^−3^) (estuarine residence).

Sr:Ca ratios in the edge of the otoliths incorporate the most recent life history information of the fish just before capture or natural death, and the Sr:Ca ratios of the otolith edge have been utilized to reconstruct the latest habitat use in fish^61,62^ including anguillid eels^23^. The Sr:Ca ratios in the otolith edges in *A. bengalensis bengalensis* and *A. bicolor bicolor* were examined in all specimens.

### Statistical analysis

The differences in the average Sr:Ca ratio for the values between different phases outside the elver mark and for the values between freshwater residence and estuarine residence at each site were examined using Student’s t-test. Differences among sites for comparisons of the average Sr:Ca ratios outside of the elver mark and the average Sr:Ca ratios in the otolith edge were examined using one-way analysis of variance (ANOVA) with Tukey HSD post hoc test for multiple comparisons.
